# A simplified morphological classification scheme for pyramidal cells in six layers of primary somatosensory cortex of juvenile rats

**DOI:** 10.1016/j.ibror.2018.10.001

**Published:** 2018-10-11

**Authors:** Yun Wang, Min Ye, Xiuli Kuang, Yaoyao Li, Shisi Hu

**Affiliations:** a School of Optometry & Ophthalmology, Wenzhou Medical University, Wenzhou, Zhejiang, P. R. China; b Allen Institute for Brain Science, Seattle, WA, USA

**Keywords:** Somatosensory cortex, Cortical layers, Cell types, Pyramidal cells, Dendrites, Apical dendrites

## Abstract

The majority of neurons in the neocortex are excitatory pyramidal cells (PCs). Many systematic classification schemes have been proposed based the neuronal morphology, the chemical composition, and the synaptic connectivity, *etc*. Recently, a cortical column of primary somatosensory cortex (SSC) has been reconstruction and functionally simulated (Markram et al., 2015). Putting forward from this study, here we proposed a simplified classification scheme for PCs in all layers of the SSC by mainly identifying apical dendritic morphology based on a large data set of 3D neuron reconstructions. We used this scheme to classify three types in layer 2, two in layer 3, three in layer 4, four in layer 5, and six types in layer 6. These PC types were visually distinguished and confirmed by quantitative differences in their morphometric properties. The classes yielded using this scheme largely corresponded with PC classes that were defined previously based on other neuronal and synaptic properties such as long-range projects and synaptic innervations, further validating its applicability. Therefore, the morphology information of apical dendrites is sufficient for a simple scheme to classify a spectrum of anatomical types of PCs in the SSC.

## Highlights

• A simplified classification scheme for PCs by identifying apical dendritic morphology.
• This classification scheme yielded 19 PC types cross all 6 layers of juvenile rat SSC.
• Three aims are:
• to introduce a simplified classification scheme for the PCs in different layers of SSC.
• to present a full spectrum of various PCs based on a large amount of neurons reconstructed from SSC.
• to reveal the structural organizing principles of PCs in different layers in a quantitative way.

## Introduction

Pyramidal cells (PC; also termed principal cells) are the major excitatory neuron type in the cerebral cortex and represent 70–85% of all neurons in the mammalian cortex (DeFelipe and Farinas, 1992; Markram et al. 2015). With rare exceptions, PCs are the only projection neurons of the cerebral cortex (Cajal, 1911; Valverde, 1986; DeFelipe and Jones, 1992). The generic anatomy of PCs in the neocortex is characterized by a pyramidal soma, two distinct dendritic domains emanating from the base and apex of the soma (basal and apical dendrites, respectively), and a single axon projecting long distances targeting other brain regions while proximally to the soma emerging out several collateral branches that further bifurcate and arborize within the neocortex. Basal dendrites fan out around the soma while the apical dendrites ascend toward the pia, in many cases giving off oblique dendrites en route and terminating in a tuft of dendrites in layer 1 or other layers. Both basal and apical dendrites typically bear a high density of spines except of occasional atypical ones (DeFelipe and Jones, 1992; Spruston, 2008a,b). The single axon branches minor collaterals profusely within the layer of origin, across neighboring layers and also projecting horizontally with varied distances forming a cluster cross multiple layers. At the extremes, some PCs have only local collaterals without extrinsic connections while some neurons may have mainly extrinsic projections with a few or no local collaterals (see review, (Rockland, 2013). The main axons of typical PCs projects long distances targeting single or multiple cortical and subcortical regions in the ipsilateral and/or contralateral hemispheres, such as the thalamus, superior colliculus, pontine nuclei, pretectal area, striatum, and contralateral cortex (Ramaswamy and Markram, 2015).

While it has been well established that PCs generally differ in their overall size and length of the apical and basal dendrites, the stereotypical arborization of an apical dendrite oversimplifies much of the diversification within each layer (Elston et al., 1997; Jacobs et al., 2001; Markram et al., 2015; Rojo et al.,2016). For example, apical dendrites can be thin or thick and may or may not reach layer 1, do not always form a tuft and some apical dendrites from the infragranular layer only project as far as layer 4 where they may or may not form a tuft. Layer 6 PCs are the most diversified with some apical dendrites projecting horizontally along the layer and even “upside down” with their apical dendrites projecting towards the white matter. Apical dendrites impart unique functional properties to PCs and form the basis for the generation of key synaptic and active events such as back propagating action potentials, Ca^2+^ spikes that propagate from their dendritic initiation sites to the soma, and integrating synaptic inputs from different cortical layers along a spectrum of temporal coincidence windows (Larkum et al., 1999; Larkum et al., 2001; Poirazi and Mel, 2001; Schaefer et al., 2003; Spruston, 2008a,b; Sakmann, 2017). The terminal tuft formed at the end of the apical dendrite is electrotonically remote and expresses different concentrations of ion channels and probably also receptors (Harnett et al., 2015), enabling local events such as persistent Ca^2+^ spikes by strong distal synaptic input (Amitai et al., 1993; Schiller et al., 1995; Schiller et al., 1997; Helmchen et al., 1999; Migliore and Shepherd, 2005) or by distributed synchronous input onto different tuft branches (Larkum et al., 2009). This regenerative activity appears to be important for binding top-down (from association areas) and bottom-up streams of input (from primary sensory and motor areas) to the neocortex that could shape the output firing pattern of PCs (Markram et al., 1995; Stuart et al., 1997; Larkum et al., 2001). PCs that can be distinguished by the morphology of their apical dendrites also often show different firing patterns and seem to form distinct synaptic sub-networks within and across the layers (Wang et al., 2006; Feldmeyer, 2012). The apical dendrites of PCs display electric resonance, which can amplify the intensity and duration of electrical activity of a neuron over a specific frequency range, impact local field potentials and hence the resulting EEG (Miller, 2007) and seems to contribute to attention mechanisms (LaBerge and Kasevich, 2013).

Generally, PCs of the same morphological type have largely the same distal targeting regions as revealed by the studies on projections of PCs mainly from infragranular layers of the neocortex (O’Leary and Koester, 1993; Veinante et al., 2000; Thomson, 2010). Their remote targets (cortical, subcortical, ipsilateral and contralateral) are genetically determined early on during differentiation and prior to the migration of the neurons to their destination layers (O’Leary and Koester, 1993; Thomson, 2010), similar to intrinsic mechanisms to determine basal dendritic field structure by the area locating the somata (Elston and Rosa, 2006). Finer analyses of their axonal and dendritic arborization, particularly their apical dendrites, suggest an association between dendritic features and differences in their target projections (Larkman and Mason, 1990; Koester and O’Leary, 1992; Kim and Connors, 1993; Kasper et al., 1994a; Franceschetti et al., 1998; Gao and Zheng, 2004; Larsen and Callaway, 2006; Morishima and Kawaguchi, 2006; Kumar and Ohana, 2008; Marx and Feldmeyer, 2012). Specific dendritic features, mainly those of apical dendrites, also correlate with how the local axon arborizes (Larsen and Callaway, 2006; Larsen et al., 2007).

A number of systematic classification schemes have been proposed based the size and shape of the apical dendrite as well as the soma locations, the axonal projection, the chemical composition, connectivity, *etc*. (van Brederode and Snyder, 1992; Kasper et al., 1994a,b; Zhang and Deschenes, 1997; Lubke et al., 2000; van Brederode et al.,2000; Lubke et al., 2003; Staiger et al.,2004; Schubert et al., 2006; Kumar and Ohana, 2008; Chen et al., 2009; Oberlaender et al., 2012; Steger et al., 2013; Kim et al.,2015; Markram et al., 2015). Especially, a great progress has been made in the reconstruction and simulation of a cortical column of primary somatosensory cortex (SSC) (Markram et al., 2015). Putting forward from this study, here we proposed a simplified classification scheme for PCs in all layers of SSC mainly by identifying apical dendritic morphology based on a large dataset of 3D neuron reconstruction. By referring previous studies, mainly on primary sensory cortices, reasonable correlations have been explored between PCs classified according to the simplified scheme and their long-distance projections and other neuronal and synaptic dynamic features.

## Methods

From the Blue Brain Project (BBP) databank (https://bbp.epfl.ch/nmc-portal), 471 pyramidal neurons were obtained, which were the neurons originally filled and stained with biocytin following whole-cell patch-clamp recordings and reconstructed with Neurolucida system (MBF Bioscience, USA) from all layers of the somatosensory cortex (SSC) in 300 μm thick brain slices of 14 to 18 days old Wistar rats (Markram et al., 1997; Gupta et al., 2000; Wang et al., 2002; Wang et al.,2004; Wang et al., 2006; Markram et al., 2015). The numbers of studied neurons were 1 in layer 1, 43 in layer 2, 44 in layer 3, 89 in layer 4, 161 in layer 5, 133 in layer 6. This dataset was considered as the most systematic morphological dataset so far including different excitatory neuronal types from all 6 cortical layers of rat SSC, which were collected under a consistent experimental condition.

The classification was carried out by subjective observation of morphological features and by combining the quantitative analysis of studied neurons, mainly their apical dendrites. Meanwhile, features of basal dendrites and local axons were also referred. This scheme has yielded three types in layer 2, two in layer 3, three in layer 4, four in layer 5, and six types in layer 6 (Table 1). Most of the PC types have been classified in a recently published work, in which the validation of the subjectively classified cell types have been made with an objective method of supervised hierarchical clustering with feature selection (Markram et al., 2015). In the current study by adding 164 newly reconstructed excitatory neurons (including 1 PC in layer 1), the classification of PCs were further refined, making up for the insufficiencies in datasets for some neuron types in the former study. While the neuron types in layers 4 and 5 were unchanged but renamed in a better systematic way throughout all 6 layers, the formerly pooled L2/3 PCs were refined into five subtypes (three in layer 2 and two in layer 3), and a narrow PC (*i.e*., L6_TPC:C) and a L6_HPC were clearly defined in layer 6. This scheme has lead to 19 excitatory cell types across 6 layers of the SSC instead of 13 excitatory cell types described in the former study. Although the morphology scheme was simplified by focusing on the most representative features of an excitatory cell type, the spectrum of cell types was not narrowed down and the neuronal diversity was even enriched in terms of morphological types of neurons in the SSC.

**Table 1:** Morphological classification of excitatory neurons in the SSC of juvenile rats.

layer	PC type	PC subtype	full name	main apical features	used name in publications
layer 2	L2_TPC	L2_TPC:A	Layer 2 Tufted PC_A	tufted, late bifurcating, small tuft	L2/3 PC
		L2_TPC:B	Layer 2 Tufted PC_B	tufted, early bifurcating, broad tuft	L2/3 PC
	L2_IPC		Layer 2 Inverted PC	inverted, later bifurcating, small tuft	

layer 3	L3_TPC	L3_TPC:A	Layer 3 Tufted PC_A	tufted, late bifurcating, multiple obliques	L2/3_PC
		L3_TPC:B	Layer 3 Tufted PC_B	tufted, late bifurcating, no/a few obliques	L2/3_PC

layer 4	L4_TPC		Layer 4 Tufted PC	tufted, late bifurcating, small tuft	L4_PC
	L4_UPC		Layer 4 Untufted PC	untufted PC	L4_SP (star PC)
	L4_SSC		Layer 4 Spiny Stellate Cell	no apical clearly outlined	L4_SS (stellate cell)

layer 5	L5_TPC	L5_TPC:A	Layer 5 Tufted PC_A	tufted, late bifurcating, broad tuft	L5_TTPC (thick tufted PC, simple PC)
		L5_TPC:B	Layer 5 Tufted PC_B	tufted, early bifurcating, broad tuft	L5_TTPC (thick tufted PC, complex PC)
		L5_TPC:C	Layer 5 Tufted PC_C	tufted, late bifurcating, small tuft	L5_STPC (slender PC)
	L5_UPC		Layer 5 Untufted PC	untufted	L5_UTPC (untufted PC)

layer 6	L6_TPC	L6_TPC:A	Layer 6 Tufted PC_A	tufted, late bifurcating, small tuft	L6_TPC_L1 and L6_TPC_L4
		L6_TPC:C	Layer 6 Tufted PC_C	tufted, narrow, late bifurcating, small tuft	L6 narrow PC
	L6_UPC		Layer 6 Untufted PC	untufted	L6_UTPC
	L6_IPC		Layer 6 Inverted PC	inverted, later bifurcating, small tuft	L6 inverted PC
	L6_BPC		Layer 6 Bipolar PC	1 upward apical and 1 downward apical	L6 bipolar PC
	L6_HPC		Layer 6 Horizontal PC	horizontal, long segments	L6 horizontal PC

**Fig. 1:**
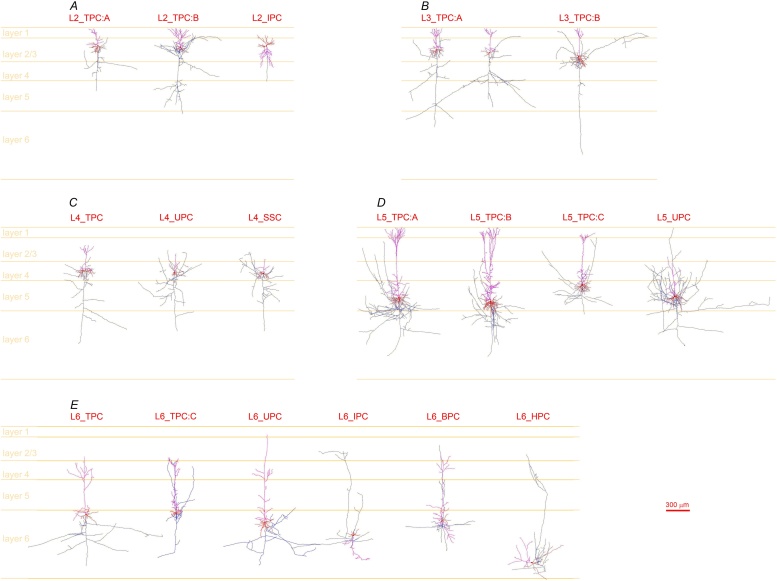
**Subjective classification of PCs in the six layers of somatosensory cortex of juvenile rats.** The classification was performed simply based on the morphological features of the apical dendrites: three types in layer 2 (A), two types in layer 3 (B), three types in layer 4 (C), four types in layer 5 (D), and six types in layer 6 (E). Reconstructed with Neurolucida system (MBF Bioscience, USA) from biocytin-filled neurons in 300 μm thick rat brain slices, an example PC represented each PC type (L3_TPC:A had two example cells showing different axon branching patterns in layer 4). Those having axonal clusters across multiple columns had been largely severed (Boudewijns, Kleele et al. 2011), leaving many collateral cutting segments attached to a main axonal stem that projects down towards white matter. Note: apical dendrites in purple, basal dendrites and somata in red, axons in dark blue.

The reconstructed neurons were quantitative analysed in multiple measurements of somata, basal and apical dendrites and axons with a software called Neuroexplorer (MBF Bioscience, USA), from which a battery of morphological parameters were obtained as the following (see in Table 2, Table 3, Table 4, Table 5, Table 6, Table 7): Soma size was presented as the *Area* and *Perimeter* of a soma that was traced at its maximal diameter. For the branching structures, the apical dendrite and the axon were defined as single trees while basal dendrites were defined as a dendritic cluster consisting of multiple trees depending upon their emerging sites from a soma. The *Max horizontal/vertical extend* was the maximal horizontal/vertical measurement of an apical/axonal tree or basal dendritic trees when the neuron was oriented perpendicular to the pia. The *Length* and *Surface* and *Volume* were respectively the total length, surface area and volume of a traced tree or a cluster. A segment is the section between two nodes or between a node and an end point or a starting point from soma. *Seg#* was the total number of all branches of a traced tree or a cluster. The *Seg length*, *Seg surface* and *Seg volume* were respectively the average length, surface area and volume of total segments of a traced tree or a cluster. For the basal dendrites, the *Den#* was, on average, the number of basal dendritic trees per neuron, and the *Tree length* was an average length of multiple basal dendritic trees. As the primary branch emanating from the soma was defined as order 1, the *Max order* was the maximal branching order of a traced apical or an axonal tree or basal dendritic trees while the *Mean order* was the average max branching order of individual trees of a basal dendritic cluster. *Tortuosity* was the ratio of the length of each branch and the straight distance between the two nodes that defined the branch. The branch angle analysis was based on averaging all angles formed in an axonal or apical tree or basal dendritic trees of a neuron and the angle measurement was reported at degrees in four different ways. *Planar angle* was the angle formed by vectors that pass through end-points of the segments forming the angle; *Local angle* was the angle formed by the intersection of the lines passing through the points closest to the node; *Local spline angle* was similar to local angle but the segments near the node have been smoothed using cubic apline; *Max angle* was defined for segments that end at nodes, which was the maximum value of the planar angles of the daughter segments (*i.e*., the other segments that are attached to the node). In addition, the average number of oblique dendrites of apical dendrites was termed *Oblique#*, and the average distribution of boutons on an axonal tree was presented as the *Bouton density*. According to the distance close or distal to soma, an apical dendrite was divided as *proximal* and *distal* parts at their middle points for a proper description of branching locations of oblique and tuft dendrites respectively. Considering the fact that axonal collaterals of most PCs filled in slices have been severed to nearly 90% or even more (Boudewijns et al., 2011), the bias in presenting data, especially of axons, have to be noticed. Relevant results were counted conditionally for the *in vitro* preparation using brain slices. Although the *in-vitro* preparation also influenced the dendrites, incomplete dendritic trees were only composed of a minor part, which would be insufficient to influence the presentation of major dendritic features of a neuron.

**Table 2:** Quantitative analysis of PCs in layer 2 of rat SSC.

		L2_TPC:A	L2_TPC:B	L2_IPC	*t*-test
		(n = 6)	(n = 33)	(n = 4)	TPC:A *vs*. TPC:B
Soma	Perimeter(μm)	56 ±2	59 ±2	**55** ±**4**	ns
	Area(μm²)	161 ±19	206 ±14	180 ±17	ns

Basal Dendrites	Max horizontal extend (μm)	179 ± 18	189 ±6	202 ±30	ns
	Max vertical extend (μm)	139 ±32	168 ±7	200±23	ns
	Den#	5 ± 0.9	5±0.3	4±1.2	ns
	Length(μm)	1483 ±303	2004 ±140	1738 ±352	ns
	Surface(μm²)	3103 ±821	4602±445	3955 ±702	ns
	Volume(μm³)	630 ±205	1099 ±156	894 ±199	ns
	Tree length (μm)	406 ±124	447 ±39	449 ±103	ns
	Seg length (μm)	46 ±4	45 ±2	49 ±7	ns
	Seg surface (μm²)	92 ±12	99 ±6	112 ±16	ns
	Seg volume (μm³)	18 ±3	23 ±3	26 ±6	ns
	Seg#	32 ±5	45 ±3	38 ±10	< 0.05
	Tortuosity	1.24 ±0.04	1.26 ±0.01	1.22 ±0.03	ns
	Max order	5 ±0.4	6 ±0.3	6 ±0.9	ns
	Mean order	3 ±0.4	4 ±0.2	4 ±0.4	ns
	Max angles	50 ±3	54 ±1	50 ±5	ns
	Planar angle	38 ±2	40 ±1	37 ±4	ns
	Local angle	55 ±3	57 ±1	58 ±4	ns
	Local spline angle	48 ±3	51 ±1	53 ±3	ns

Apical Dendrites	Max horizontal extend (μm)	191 ±30	247 ±12	222 ±28	ns
	Max vertical extend (μm)	198 ±24	237 ±8	229 ±18	ns
	Length(μm)	1514 ±272	2666 ± 147	2659 ±229	< 0.05
	Surface(μm²)	3439 ±797	6384 ±571	5750 ±890	< 0.05
	Volume(μm³)	794 ±223	1712 ±254	1305 ±287	< 0.05
	Seg length (μm)	46 ±4	59 ±3	54 ±4	< 0.05
	Seg surface (μm²)	102 ±13	140 ±12	115 ±10	< 0.05
	Seg volume (μm³)	23 ±4	37 ±5	26 ±4	< 0.05
	Seg#	33 ±7	50 ±5	49 ±4	< 0.05
	Tortuosity	1.21 ±0.03	1.24 ±0.01	1.22 ±0.03	ns
	Max order	9 ±0.8	11 ±0.5	12 ±0.3	ns
	Max angles	53 ±5	52 ±1	53 ±4	ns
	Planar angle	39 ±3	39 ±1	39 ±4	ns
	Local angle	58 ±2	58 ±1	58 ±4	ns
	Local spline angle	49 ±3	51 ±1	54 ±4	ns
	Oblique#	6.5 ±1.1	5.0 ±0.4	8.5 ±1.9	< 0.05

Axon	Max horizontal extend (μm)	410 ±127	779 ±89	467 ±318	< 0.05
	Max vertical extend (μm)	494 ±116	914 ±93	650 ±294	< 0.05
	Length(μm)	2315 ±851	6404 ±816	4043 ±2462	< 0.05
	Surface(μm²)	2106 ±582	3748 ±422	2476 ±1365	< 0.05
	Volume(μm³)	197 ±50	235 ±24	160 ±73	ns
	Seg length (μm)	84 ±12	108 ±13	217 ±151	ns
	Seg surface (μm²)	85 ±10	86 ±22	219 ±190	ns
	Seg volume (μm³)	9 ±1	8 ±3	22 ±21	ns
	Seg#	25 ±6	64 ±7	38 ±19	< 0.05
	Tortuosity	1.16 ±0.02	1.18 ±0.01	1.20 ±0.05	ns
	Max order	8 ±1.4	12 ±1.0	8 ±3.5	< 0.05
	Max angles	81 ±6	73 ±2	78 ±7	ns
	Planar angle	51 ±3	47 ±1	53 ±5	ns
	Local angle	63 ±3	59 ±1	63 ±4	ns
	Local spline angle	56 ±4	53 ±1	57 ±4	ns
	Boton density (#/100 μm)	17 ±3	18 ±1	21 ±2	Ns

**Table 3:** Quantitative analysis of PCs in layer 3 of rat SSC.

		L3_TPC:A	L3_TPC:B	*t*-test
		(n = 35)	(n = 9)	TPC:A *vs*. TPC:B
Soma	perometer and	58 ±2	53 ±3	ns
	Area(μm²)	195 ±10	160 ±23	ns

Basal Dendrites	Max horizontal extend (μm)	230 ±7	230 ±14	ns
	Max vertical extend (μm)	183 ±9	190 ±18	ns
	Den#	5±0.2	5±0.2	ns
	Length(μm)	2410 ±136	2393 ±273	ns
	Surface(μm²)	5923 ±489	5298 ±1043	ns
	Volume(μm³)	1475 ±189	1183 ±380	ns
	Tree length (μm)	467 ±24	499 ±51	ns
	Seg length (μm)	53 ±2	55 ±3	ns
	Seg surface (μm²)	129 ±8	115 ±10	ns
	Seg volume (μm³)	31 ±4	24 ±5	ns
	Seg#	46 ±2	44 ±6	ns
	Tortuosity	1.26 ±0.01	1.27 ±0.03	ns
	Max order	6±0.2	6±0.4	ns
	Mean order	4±0.1	4±0.3	ns
	Max angles	54 ±1	57 ±3	ns
	Planar angle	41 ±1	43 ±2	ns
	Local angle	59 ±1	59 ±2	ns
	Local spline angle	52 ±1	53 ±2	ns

Apical Dendrites	Max horizontal extend (μm)	190 ±7	127 ±14	< 0.05
	Max vertical extend (μm)	338 ±11	377 ±30	ns
	Length(μm)	2191 ±114	1157 ±108	< 0.05
	Surface(μm²)	5594 ±405	2882 ±479	< 0.05
	Volume(μm³)	1499 ±160	731 ±199	< 0.05
	Seg length (μm)	58 ±2	74 ±11	ns
	Seg surface (μm²)	148 ±8	171 ±25	ns
	Seg volume (μm³)	39 ±4	39 ±6	ns
	Seg#	39 ±2	19 ±4	< 0.05
	Tortuosity	1.24 ±0.01	1.23 ±0.03	ns
	Max order	11 ±0.4	6 ±0.8	< 0.05
	Max angles	53 ±1	50 ±3	ns
	Planar angle	38 ±1	35 ±2	ns
	Local angle	58 ±1	53 ±2	< 0.05
	Local spline angle	50 ±1	44 ±2	< 0.05
	Oblique#	4.9 ±0.3	2.3 ±0.4	< 0.05

Axon	Max horizontal extend (μm)	735 ±71	753 ±153	ns
	Max vertical extend (μm)	859 ±75	761 ±162	ns
	Length(μm)	6176 ±695	5269 ±1246	ns
	Surface(μm²)	4344 ±384	3563 ±910	ns
	Volume(μm³)	323 ±34	279 ±79	ns
	Seg length (μm)	100 ±6	97 ±13	ns
	Seg surface (μm²)	84 ±9	75 ±12	ns
	Seg volume (μm³)	7 ±1	9 ±4	ns
	Seg#	64 ±8	52 ±11	ns
	Tortuosity	1.16 ±0.01	1.19 ±0.03	ns
	Max order	11 ±0.7	10 ±1.1	ns
	Max angles	73 ±1	77 ±3	ns
	Planar angle	47 ±1	49 ±2	ns
	Local angle	61 ±1	60 ±1	ns
	Local spline angle	53 ±1	55 ±1	ns
	Boton density (#/100 μm)	20 ±1	19 ±1	ns

**Table 4:** Quantitative analysis of excitaory cells in layer 4 of rat SSC.

		L4_TPC	L4_UPC	L4_SSC		t-test	
		(n = 44)	(n = 33)	(n = 12)	TPC *vs*. UPC	TPC *vs*. SSC	UPC *vs*. SSC
Soma	Perimeter(μm)	64 ±2	60 ±2	57 ±2	ns	< 0.05	ns
	Area(μm²)	248 ±15	225 ±14	180 ±7	ns	< 0.05	< 0.05

Basal Dendrites	Max horizontal extend (μm)	263 ±10	242 ±13	272±32	ns	ns	ns
	Max vertical extend (μm)	212 ±10	219 ±14	203 ±22	ns	ns	ns
	Den#	6±0.2	5±0.3	5±0.3	< 0.05	< 0.05	ns
	Length(μm)	2387 ±155	1899±156	2141 ±149	< 0.05	ns	ns
	Surface(μm²)	5917 ±481	4805±444	4742 ±357	ns	ns	ns
	Volume(μm³)	1550 ±189	1303 ±173	1066 ±119	ns	< 0.05	ns
	Tree length (μm)	435 ±29	435 ±45	469 ±46	ns	ns	ns
	Seg length (μm)	66 ±2	64 ±4	69 ±7	ns	ns	ns
	Seg surface (μm²)	164 ±10	167 ±14	155 ±17	ns	ns	ns
	Seg volume (μm³)	43 ±5	46 ±6	35 ±5	ns	ns	ns
	Seg#	38 ±3	32 ±3	33 ±3	ns	ns	ns
	Tortuosity	1.27 ±0.01	1.25 ±0.01	1.32 ±0.02	ns	< 0.05	< 0.05
	Max order	5±0.2	5±0.3	5±0.3	ns	ns	ns
	Mean order	3±0.2	3±0.2	3±0.2	ns	ns	ns
	Max angles	56 ±1	57 ±1	55±3	ns	ns	ns
	Planar angle	42 ±1	42 ±1	40 ±2	ns	ns	ns
	Local angle	58 ±1	60 ±1	58 ±3	ns	ns	ns
	Local spline angle	52 ±1	52 ±1	52 ±2	ns	ns	ns

Apical Dendrites	Max horizontal extend (μm)	210 ±10	180 ±8	169 ±14	< 0.05	< 0.05	ns
	Max vertical extend (μm)	547 ±23	451 ±23	191 ±14	< 0.05	< 0.05	< 0.05
	Length(μm)	2077 ±108	1496 ±76	1023 ±140	< 0.05	< 0.05	< 0.05
	Surface(μm²)	5552 ±359	4138 ±294	2526 ±386	< 0.05	< 0.05	< 0.05
	Volume(μm³)	1573 ±157	1224 ±132	657 ±113	ns	< 0.05	< 0.05
	Seg length (μm)	75 ±3	75 ±4	65 ±6	ns	ns	ns
	Seg surface (μm²)	202 ±12	212 ±18	165 ±23	ns	ns	ns
	Seg volume (μm³)	57 ±6	64 ±8	45 ±9	ns	ns	ns
	Seg#	30 ±2	22 ±2	18 ±3	< 0.05	< 0.05	ns
	Tortuosity	1.24 ±0.01	1.22 ±0.03	1.31 ±0.02	ns	< 0.05	< 0.05
	Max order	10±1	8±0.4	6±0.7	< 0.05	< 0.05	< 0.05
	Max angles	56 ±1	60 ±2	64 ±4	< 0.05	ns	ns
	Planar angle	39 ±1	41 ±1	45 ±2	ns	< 0.05	ns
	Local angle	57 ±1	58 ±1	56±3	ns	ns	ns
	Local spline angle	50 ±1	51 ±1	53 ±2	ns	ns	ns
	Oblique#	6.4 ±0.5	6.4 ±0.4	4.5 ±0.5	ns	< 0.05	< 0.05

Axon	Max horizontal extend (μm)	724 ±62	758 ±82	638 ±62	ns	ns	ns
	Max vertical extend (μm)	1011 ±61	1058 ±79	1103 ±90	ns	ns	ns
	Length(μm)	5713 ±567	6237 ±760	7838 ±894	ns	< 0.05	ns
	Surface(μm²)	3957 ±404	3605 ±369	5967 ±672	ns	< 0.05	< 0.05
	Volume(μm³)	302 ±37	230 ±28	461 ±83	ns	ns	< 0.05
	Seg length (μm)	113 ±5	110 ±6	93 ±4	ns	< 0.05	< 0.05
	Seg surface (μm²)	84 ±7	68 ±5	76 ±10	< 0.05	ns	ns
	Seg volume (μm³)	7 ±1	5 ±1	6 ±1	< 0.05	ns	ns
	Seg#	50 ±5	55 ±5	84 ±9	ns	< 0.05	< 0.05
	Tortuosity	1.19 ±0.01	1.19 ±0.01	1.20 ±0.01	ns	ns	ns
	Max order	11±0.5	11±0.5	12±0.7	ns	ns	ns
	Max angles	81 ±2	78 ±1	72 ±3	ns	< 0.05	< 0.05
	Planar angle	52 ±1	51 ±1	47 ±1	ns	< 0.05	< 0.05
	Local angle	61 ±1	58 ±1	57 ±1	< 0.05	< 0.05	ns
	Local spline angle	55 ±1	52 ±1	51 ±1	< 0.05	< 0.05	ns
	Boton density (#/100 μm)	19 ±1	22 ±1	18 ±1	< 0.05	ns	< 0.05

**Table 5:** Quantitative analysis of PCs in layer 5 of rat SSC.

		L5_TPC:A	L5_TPC:B	L5_TPC:C	L5_UPC	t-test					
		(n = 60)	(n = 38)	(n = 33)	(n = 30)	TPC:A *vs*. TPC:B	TPC:A *vs*. TPC:C	TPC:A *vs*. UPC	TPC:B *vs*. TPC:C	TPC:B *vs*. UPC	TPC:C *vs*. UPC
Soma	Perimeter(μm)	83 ±2	85 ±2	71 ±4	68 ±3	ns	< 0.05	< 0.05	< 0.05	< 0.05	ns
	Area(μm²)	450 ±17	471±17	317±33	326 ±29	ns	< 0.05	< 0.05	< 0.05	< 0.05	ns

Basal Dendrites	Max horizontal extend (μm)	310 ±8	308 ±10	290 ±15	265 ±12	ns	ns	< 0.05	ns	< 0.05	ns
	Max vertical extend (μm)	252 ±8	268 ±12	247 ±12	274 ±20	ns	ns	ns	ns	ns	ns
	Den#	7±0.2	7±0.3	6±0.3	6±0.3	ns	< 0.05	< 0.05	< 0.05	< 0.05	ns
	Length(μm)	3882 ±184	4299±210	2642 ±203	3034±220	ns	< 0.05	< 0.05	< 0.05	< 0.05	ns
	Surface(μm²)	10,645 ±661	11,713 ±747	5689 ±477	7454 ±735	ns	< 0.05	< 0.05	< 0.05	< 0.05	< 0.05
	Volume(μm³)	2981 ±249	3285 ±285	1349±189	1889 ±264	ns	< 0.05	< 0.05	< 0.05	< 0.05	ns
	Tree length (μm)	562 ±27	617 ±34	448 ±35	500 ±30	ns	< 0.05	ns	< 0.05	< 0.05	ns
	Seg length (μm)	65 ±2	59 ±2	72 ±4	69 ±6	< 0.05	< 0.05	ns	< 0.05	ns	ns
	Seg surface (μm²)	179 ±9	158 ±7	160 ±11	171 ±20	ns	ns	ns	ns	ns	ns
	Seg volume (μm³)	52 ±5	44 ±3	39 ±5	44 ±7	ns	ns	ns	ns	ns	ns
	Seg#	61 ±3	75 ±3	40 ±4	47 ±3	< 0.05	< 0.05	< 0.05	< 0.05	< 0.05	ns
	Tortuosity	1.28 ±0.01	1.25 ±0.01	1.28 ±0.01	1.28 ±0.01	< 0.05	ns	ns	ns	ns	ns
	Max order	6±0.2	6±0.2	5±0.3	5±0.2	ns	< 0.05	ns	< 0.05	< 0.05	ns
	Mean order	4±0.1	4±0.2	3±0.2	4±0.1	< 0.05	< 0.05	ns	< 0.05	< 0.05	< 0.05
	Max angles	55 ±1	57 ±1	53 ±1	57 ±2	ns	ns	ns	ns	ns	ns
	Planar angle	41 ±1	43 ±1	40 ±1	43 ±1	ns	ns	ns	ns	ns	ns
	Local angle	58 ±1	58 ±1	57 ±2	60 ±1	ns	ns	ns	ns	ns	ns
	Local spline angle	51 ±1	51 ±1	50 ±2	53 ±1	ns	ns	ns	ns	ns	ns

Apical Dendrites	Max horizontal extend (μm)	356 ±10	350 ±13	252 ±11	216 ±10	ns	< 0.05	< 0.05	< 0.05	< 0.05	< 0.05
	Max vertical extend (μm)	857 ±20	948 ±18	767 ±33	596 ±28	< 0.05	< 0.05	< 0.05	< 0.05	< 0.05	< 0.05
	Length(μm)	7280±267	8512 ±290	3522 ±246	2787 ±225	< 0.05	< 0.05	< 0.05	< 0.05	< 0.05	< 0.05
	Surface(μm²)	22,959±1117	26,835 ±1407	9177 ±884	7360 ±773	< 0.05	< 0.05	< 0.05	< 0.05	< 0.05	ns
	Volume(μm³)	8957±664	10,270 ±913	3151±549	2232 ±328	ns	< 0.05	< 0.05	< 0.05	< 0.05	ns
	Seg length (μm)	69 ±2	66 ±3	77 ±3	75 ±5	ns	< 0.05	ns	< 0.05	ns	ns
	Seg surface (μm²)	219 ±11	201 ±10	202±17	204 ±20	ns	ns	ns	ns	ns	ns
	Seg volume (μm³)	87 ±8	73 ±5	66 ±11	63 ±9	ns	ns	< 0.05	ns	ns	ns
	Seg#	111 ±6	145 ±11	52 ±6	42 ±4	< 0.05	< 0.05	< 0.05	< 0.05	< 0.05	ns
	Tortuosity	1.28 ±0.01	1.26 ±0.01	1.27 ±0.01	1.27±0.01	ns	ns	ns	ns	ns	ns
	Max order	21 ±1	23 ±1	15 ±1	14 ±1	ns	< 0.05	< 0.05	< 0.05	< 0.05	ns
	Max angles	57 ±1	59 ±2	59 ±2	63 ±2	ns	ns	< 0.05	ns	ns	< 0.05
	Planar angle	41 ±1	43 ±1	41 ±1	43 ±1	ns	ns	ns	ns	ns	ns
	Local angle	59 ±1	58 ±1	58 ±1	58 ±1	ns	ns	ns	ns	ns	ns
	Local spline angle	52 ±1	51 ±1	51 ±1	52 ±1	ns	ns	ns	ns	ns	ns
	Oblique#	12.5 ±0.5	12.8 ±0.6	10.3 ±1.0	12.2 ±0.8	ns	ns	ns	< 0.05	ns	ns

Axon	Max horizontal extend (μm)	1003 ±64	1035 ±69	639±55	1104 ±78	ns	< 0.05	ns	< 0.05	ns	< 0.05
	Max vertical extend (μm)	1046 ±56	985 ±69	921 ±70	1107 ±61	ns	ns	ns	ns	ns	< 0.05
	Length(μm)	8704±604	9847 ±1154	5741±712	9079±923	ns	< 0.05	ns	< 0.05	ns	< 0.05
	Surface(μm²)	7680±1039	7898 ±800	3944±414	5360 ±887	ns	< 0.05	ns	< 0.05	< 0.05	ns
	Volume(μm³)	1348 ±473	1012±108	459 ±73	453 ±118	ns	ns	ns	< 0.05	< 0.05	ns
	Seg length (μm)	107 ±4	95 ±4	106 ±6	125 ±5	< 0.05	ns	< 0.05	ns	< 0.05	< 0.05
	Seg surface (μm²)	103 ±12	91 ±7	81 ±8	71 ±5	ns	ns	< 0.05	ns	< 0.05	ns
	Seg volume (μm³)	19 ±6	14 ±2	11 ±2	6 ±1	ns	ns	< 0.05	ns	< 0.05	< 0.05
	Seg#	84 ±6	99 ±10	54 ±6	75 ±9	ns	< 0.05	ns	< 0.05	ns	< 0.05
	Tortuosity	1.21 ±0.01	1.17 ±0.01	1.20 ±0.01	1.20±0.01	< 0.05	ns	ns	< 0.05	< 0.05	ns
	Max order	12±0.5	12±0.5	10±0.5	12±0.7	ns	< 0.05	ns	< 0.05	ns	< 0.05
	Max angles	70 ±1	68 ±1	76 ±2	72 ±1	ns	< 0.05	ns	< 0.05	< 0.05	ns
	Planar angle	46 ±1	45 ±1	50 ±1	47 ±1	ns	< 0.05	ns	< 0.05	< 0.05	ns
	Local angle	60 ±1	58 ±1	61 ±1	58 ±1	ns	ns	ns	ns	ns	ns
	Local spline angle	54 ±1	51 ±1	54 ±1	52 ±1	< 0.05	ns	ns	< 0.05	ns	ns
	Boton density (#/100 μm)	15 ±1	16 ±1	21 ±1	21 ±1	ns	< 0.05	< 0.05	< 0.05	< 0.05	ns

**Table 6:** Quantitative analysis of PCs in layer 6 of rat SSC.

		L6_TPC:A	L6_TPC:C	L6_UPC	L6_IPC	L6_BPC	L6_HPC	*t*-test														
		(n = 26)	(n = 18)	(n = 23)	(n = 27)	(n = 32)	(n = 7)	TPC:A *vs*. TPC:C	TPC:A *vs*. UPC	TPC:A *vs*. IPC	TPC:A *vs*. BPC	UPC vs. TPC:C	IPC *vs*. TPC:C	BPC *vs*.TPC:C	UPC *vs*. IPC	UPC *vs*. BPC	IPC *vs*.BPC	HPC *vs* TPC:A	HPC *vs* TPC:C	HPC *vs* UPC	HPC *vs* IPC	HPC *vs* BPC
Soma	Perimeter(μm)	53 ± 2	51±3	51 ±1	54 ±2	53 ±2	64±3	ns	ns	ns	ns	ns	ns	ns	ns	ns	ns	< 0.05	< 0.05	< 0.05	< 0.05	< 0.05
	Area(μm²)	184 ±12	159 ±9	179 ±8	179 ±9	185 ±13	243 ±10	ns	ns	ns	ns	ns	ns	ns	ns	ns	ns	< 0.05	< 0.05	< 0.05	< 0.05	< 0.05

Basal Dendrites	Max horizontal extend (μm)	217 ±14	161 ±7	232±15	248±26	196 ±13	407 ±85	< 0.05	ns	ns	ns	< 0.05	< 0.05	< 0.05	ns	ns	ns	< 0.05	< 0.05	ns	ns	< 0.05
	Max vertical extend (μm)	208 ±16	123 ±7	198 ±15	192 ±18	171 ±18	360 ±51	< 0.05	ns	ns	ns	< 0.05	< 0.05	< 0.05	ns	ns	ns	< 0.05	< 0.05	< 0.05	< 0.05	< 0.05
	Den#	5 ±0.3	7 ±0.4	6 ±0.3	6 ±0.3	4 ±0.3	4 ±0.7	< 0.05	ns	ns	< 0.05	ns	< 0.05	< 0.05	ns	< 0.05	< 0.05	< 0.05	< 0.05	< 0.05	< 0.05	ns
	Length(μm)	1716±128	1257±92	1857±188	1522 ±111	1174 ±103	2029 ±395	< 0.05	ns	ns	< 0.05	< 0.05	ns	ns	ns	< 0.05	< 0.05	ns	ns	ns	ns	ns
	Surface(μm²)	3039 ±282	2150±200	3500±373	2540±207	2063 ±221	4444 ±1320	< 0.05	ns	ns	< 0.05	< 0.05	ns	ns	< 0.05	< 0.05	ns	ns	ns	ns	ns	ns
	Volume(μm³)	567±71	360 ±61	699 ±98	464±63	385 ±59	1145 ±469	< 0.05	ns	ns	< 0.05	< 0.05	ns	ns	< 0.05	< 0.05	ns	ns	ns	ns	ns	ns
	Tree length (μm)	349 ±34	203 ±20	338±32	299 ±38	299 ±25	726 ±197	< 0.05	ns	ns	ns	< 0.05	< 0.05	< 0.05	ns	ns	ns	ns	< 0.05	ns	ns	ns
	Seg length (μm)	56 ±4	45 ±3	64 ±6	63 ±6	56 ±3	101±8	< 0.05	ns	ns	ns	< 0.05	< 0.05	< 0.05	ns	ns	ns	< 0.05	< 0.05	< 0.05	< 0.05	< 0.05
	Seg surface (μm²)	96 ±7	80 ±9	127±17	103 ±8	100 ±7	212 ±32	ns	ns	ns	ns	< 0.05	ns	ns	ns	ns	ns	< 0.05	< 0.05	< 0.05	< 0.05	< 0.05
	Seg volume (μm³)	18 ±2	14 ±3	27 ±6	19 ±3	19 ±3	52 ±13	ns	ns	ns	ns	< 0.05	ns	ns	ns	ns	ns	< 0.05	< 0.05	ns	< 0.05	< 0.05
	Seg#	34 ±4	28 ±2	33 ±5	27 ±2	22 ±2	20 ±3	ns	ns	ns	< 0.05	ns	ns	< 0.05	ns	ns	ns	< 0.05	< 0.05	< 0.05	ns	ns
	Tortuosity	1.27±0.01	1.34 ±0.03	1.28±0.02	1.25 ±0.01	1.27 ±0.02	1.20 ±0.02	< 0.05	ns	ns	ns	ns	< 0.05	ns	ns	ns	ns	< 0.05	< 0.05	< 0.05	< 0.05	< 0.05
	Max order	5±0.6	4±0.2	5±0.3	4±0.2	5±0.5	4±0.5	ns	ns	< 0.05	ns	ns	ns	ns	ns	ns	ns	ns	ns	ns	ns	ns
	Mean order	3±0.3	3±0.2	3±0.2	3±0.2	3±0.2	3±0.5	< 0.05	ns	< 0.05	ns	ns	ns	ns	ns	ns	ns	ns	ns	ns	ns	ns
	Max angles	61 ±2	64 ±2	57 ±2	60 ±2	57 ±2	46±2	ns	ns	ns	ns	< 0.05	ns	< 0.05	ns	ns	ns	< 0.05	< 0.05	< 0.05	< 0.05	< 0.05
	Planar angle	44 ±1	42 ±4	42 ±1	42 ±2	38 ±2	34 ±1	ns	ns	ns	< 0.05	ns	ns	ns	ns	ns	ns	< 0.05	< 0.05	< 0.05	< 0.05	ns
	Local angle	58 ±2	51 ±6	58 ±2	56 ±2	58 ±3	50 ±3	ns	ns	ns	ns	ns	ns	ns	ns	ns	ns	< 0.05	ns	< 0.05	< 0.05	< 0.05
	Local spline angle	54 ±2	47 ±5	50 ±1	48±2	50 ±2	43 ±3	ns	< 0.05	< 0.05	ns	ns	ns	ns	ns	ns	ns	< 0.05	ns	< 0.05	ns	< 0.05

Apical Dendrites	Max horizontal extend (μm)	321 ±20	189 ±11	249 ±19	312 ±22	219±17	431±83	< 0.05	< 0.05	ns	< 0.05	< 0.05	< 0.05	ns	< 0.05	ns	< 0.05	ns	< 0.05	ns	ns	< 0.05
	Max vertical extend (μm)	690±33	731 ±53	714±48	397 ±32	580 ±36	451 ±62	ns	ns	< 0.05	< 0.05	ns	< 0.05	< 0.05	< 0.05	< 0.05	< 0.05	< 0.05	< 0.05	< 0.05	ns	ns
	Length(μm)	3390 ±194	3239±210	3136±280	2612±255	2304±191	3215 ±912	ns	ns	< 0.05	< 0.05	ns	ns	< 0.05	ns	< 0.05	ns	ns	ns	ns	ns	ns
	Surface(μm²)	6120±456	6413±597	6242±605	4797±434	3955 ±283	6194 ±1206	ns	ns	< 0.05	< 0.05	ns	< 0.05	< 0.05	ns	< 0.05	ns	ns	ns	ns	ns	ns
	Volume(μm³)	1192±142	1403±245	1425±200	979 ±98	766 ±83	1472 ±289	ns	ns	ns	< 0.05	ns	ns	< 0.05	ns	< 0.05	ns	ns	ns	ns	ns	< 0.05
	Seg length (μm)	90 ±6	62 ±4	82 ±7	71 ±6	78 ±6	98 ±5	< 0.05	ns	< 0.05	ns	< 0.05	ns	< 0.05	ns	ns	ns	ns	< 0.05	ns	< 0.05	< 0.05
	Seg surface (μm²)	160 ±11	122±10	167 ±17	138 ±11	151 ±20	204 ±23	< 0.05	ns	ns	ns	< 0.05	ns	ns	ns	ns	ns	ns	< 0.05	ns	< 0.05	ns
	Seg volume (μm³)	31 ±4	26 ±4	38 ±5	31 ±4	32 ±6	51 ±10	ns	ns	ns	ns	ns	ns	ns	ns	ns	ns	ns	< 0.05	ns	ns	ns
	Seg#	43 ±5	54 ±5	42 ±5	39 ±4	34 ±4	31±7	ns	ns	ns	ns	ns	< 0.05	< 0.05	ns	ns	ns	ns	< 0.05	ns	ns	ns
	Tortuosity	1.22 ±0.02	1.28±0.02	1.25 ±0.03	1.22 ±0.03	1.23±0.01	1.20 ±0.03	ns	ns	ns	ns	ns	ns	ns	ns	ns	ns	ns	< 0.05	ns	ns	ns
	Max order	14 ±1	20±1	14 ±1	12 ±1	12 ±1	7 ±1	< 0.05	ns	ns	< 0.05	< 0.05	< 0.05	< 0.05	ns	< 0.05	ns	< 0.05	< 0.05	< 0.05	< 0.05	< 0.05
	Max angles	59 ±2	66 ±2	63±2	61 ±2	55 ±2	54 ±4	< 0.05	ns	ns	ns	ns	< 0.05	< 0.05	ns	< 0.05	< 0.05	ns	< 0.05	ns	ns	ns
	Planar angle	39 ±1	40±3	42 ±1	43 ±1	36 ±2	40 ±3	ns	ns	ns	ns	ns	ns	ns	ns	< 0.05	< 0.05	ns	ns	ns	ns	ns
	Local angle	61 ±1	55 ±4	59 ±2	63 ±2	57 ±2	56 ±4	ns	ns	ns	ns	ns	< 0.05	ns	ns	ns	< 0.05	ns	ns	ns	ns	ns
	Local spline angle	52 ±1	51 ±2	53 ±1	52 ±1	49 ±1	49 ±3	ns	ns	ns	ns	ns	ns	ns	ns	< 0.05	ns	ns	ns	ns	ns	ns
	Oblique#	9.2±0.7	14.4 ±0.9	12.9±1.0	11 ±1	8.6 ±0.6	3.7±0.8	< 0.05	< 0.05	ns	ns	ns	< 0.05	< 0.05	ns	< 0.05	< 0.05	< 0.05	< 0.05	< 0.05	< 0.05	< 0.05

Axon	Max horizontal extend (μm)	778±136	315 ±53	798 ±113	868 ±133	841±98	715 ±157	< 0.05	ns	ns	ns	< 0.05	< 0.05	< 0.05	ns	ns	ns	ns	< 0.05	ns	ns	ns
	Max vertical extend (μm)	560 ±50	628 ±102	691±87	750 ±90	691 ±56	773 ±247	ns	ns	ns	ns	ns	ns	ns	ns	ns	ns	ns	ns	ns	ns	ns
	Length(μm)	3648 ±545	2503±508	4670±753	4947 ±859	4428 ±570	4518±1480	ns	ns	ns	ns	< 0.05	< 0.05	< 0.05	ns	ns	ns	ns	ns	ns	ns	ns
	Surface(μm²)	2397 ±356	2387 ±517	3074±505	3077±467	2729 ±324	2910±821	ns	ns	ns	ns	ns	ns	ns	ns	ns	ns	ns	ns	ns	ns	ns
	Volume(μm³)	204±33	254 ±62	286 ±48	220 ±36	227 ±40	251 ±88	ns	ns	ns	ns	ns	ns	ns	ns	ns	ns	ns	ns	ns	ns	ns
	Seg length (μm)	125±14	87 ±13	106±10	115 ±11	113 ±7	112 ±16	< 0.05	ns	ns	ns	ns	ns	ns	ns	ns	ns	ns	ns	ns	ns	ns
	Seg surface (μm²)	108 ±19	91 ±14	76 ±8	89 ±15	74 ±8	79 ±13	ns	ns	ns	ns	ns	ns	ns	ns	ns	ns	ns	ns	ns	ns	ns
	Seg volume (μm³)	14±4	11 ±2	8 ±1	8 ±2	7 ±1	8 ±2	ns	ns	ns	ns	ns	ns	ns	ns	ns	ns	ns	ns	ns	ns	ns
	Seg#	39 ±8	45 ±19	51 ±12	44 ±9	42 ±5	46 ±17	ns	ns	ns	ns	ns	ns	ns	ns	ns	ns	ns	ns	ns	ns	ns
	Tortuosity	1.23±0.03	1.18±0.02	1.19 ±0.01	1.19 ±0.01	1.17±0.01	1.17 ±0.02	ns	ns	ns	ns	ns	ns	ns	ns	ns	ns	ns	ns	ns	ns	ns
	Max order	7 ±1	10 ±2	10 ±1	8 ±0.8	10 ±1	10 ±3	ns	ns	ns	< 0.05	ns	ns	ns	ns	ns	ns	ns	ns	ns	ns	ns
	Max angles	75 ±2	92 ±5	80 ±3	69 ±2	74 ±1	83 ±4	< 0.05	ns	ns	ns	ns	< 0.05	< 0.05	< 0.05	ns	ns	ns	ns	ns	ns	ns
	Planar angle	50±2	62 ±4	54 ±2	47 ±2	49 ±1	55 ±4	< 0.05	ns	ns	ns	ns	< 0.05	< 0.05	< 0.05	< 0.05	ns	ns	ns	ns	ns	ns
	Local angle	62 ±2	63 ±2	62 ±2	62 ±2	62 ±1	65 ±6	ns	ns	ns	ns	ns	ns	ns	ns	ns	ns	ns	ns	ns	ns	ns
	Local spline angle	54±1	56 ±2	53 ±1	51 ±1	55 ±1	60±6	ns	ns	< 0.05	ns	ns	< 0.05	ns	ns	ns	< 0.05	ns	ns	ns	ns	ns
	Boton density (#/100 μm)	20±2	17 ±2	19 ±1	19 ±2	20 ±1	22 ±1	ns	ns	ns	ns	ns	ns	ns	ns	ns	ns	ns	< 0.05	ns	ns	ns

**Table 7:** Quantitative comparison between L2_IPCs and L6_IPCs of rat SSC.

		L2_IPC	L6_IPC	t-test
		(n = 4)	(n = 27)	L2_IPC *vs*. L6_IPC
Soma	Perimeter(μm)	55 ±4	54 ±2	ns
	Area(μm²)	180 ±17	179 ±9	ns

Basal Dendrites	Max horizontal extend (μm)	202 ±30	248 ±26	ns
	Max vertical extend (μm)	200 ±23	192 ±18	ns
	Den#	4±1.2	6±0.3	ns
	Length(μm)	1738 ±352	1522 ±111	ns
	Surface(μm²)	3955 ±702	2540 ±207	ns
	Volume(μm³)	894 ±199	464 ±63	ns
	Tree length (μm)	449 ±103	299 ±38	ns
	Seg length (μm)	49 ±7	63 ±6	ns
	Seg surface (μm²)	112 ±16	103 ±8	ns
	Seg volume (μm³)	26 ±6	19 ±3	ns
	Seg#	38 ±10	27 ±2	ns
	Tortuosity	1.22 ±0.03	1.25 ±0.01	ns
	Max order	6±0.9	4±0.2	ns
	Mean order	4±0.4	3±0.2	< 0.05
	Max angles	50 ±5	60 ±2	ns
	Planar angle	37 ±4	42 ±2	ns
	Local angle	58 ±4	56 ±2	ns
	Local spline angle	53 ±3	48 ±2	ns

Apical Dendrites	Max horizontal extend (μm)	222 ±28	312 ±22	< 0.05
	Max vertical extend (μm)	229 ±18	397 ±32	< 0.05
	Length(μm)	2659 ±229	2612 ±255	ns
	Surface(μm²)	5750 ±890	4797 ±434	ns
	Volume(μm³)	1305 ±287	979 ±98	ns
	Seg length (μm)	54 ±4	71 ±6	< 0.05
	Seg surface (μm²)	115 ±10	138 ±11	ns
	Seg volume (μm³)	26 ±4	31 ±4	ns
	Seg#	49 ±4	39 ±4	ns
	Tortuosity	1.22 ±0.03	1.22 ±0.03	ns
	Max order	12 ±0.3	12 ±1	ns
	Max angles	53 ±4	61 ±2	ns
	Planar angle	39 ±4	43 ±1	ns
	Local angle	58 ±4	63 ±2	ns
	Local spline angle	54 ±4	52 ±1	ns
	Oblique#	9 ±2	11 ±1	ns

Axon	Max horizontal extend (μm)	467 ±318	868 ±133	ns
	Max vertical extend (μm)	650 ±294	750 ±90	ns
	Length(μm)	4043 ±2462	4947 ±859	ns
	Surface(μm²)	2476 ±1365	3077 ±467	ns
	Volume(μm³)	160 ±73	220 ±36	ns
	Seg length (μm)	217 ±151	115 ±11	ns
	Seg surface (μm²)	219 ±190	89 ±15	ns
	Seg volume (μm³)	22 ±21	8 ±2	ns
	Seg#	38 ±19	44 ±9	ns
	Tortuosity	1.20 ±0.05	1.19 ±0.01	ns
	Max order	8 ±3	8 ±0.8	ns
	Max angles	78 ±7	69 ±2	ns
	Planar angle	53 ±5	47 ±2	ns
	Local angle	63 ±4	62 ±2	ns
	Local spline angle	57 ±4	51 ±1	ns
	Boton density (#/100 μm)	21 ±2	19 ±2	ns

For the statistical analysis, un-paired student *t*-test was used to compare individual quantitative morphological parameters of single neurons between different types. The significance level for comparison was P ≤ 0.05 (Table 2, Table 3, Table 4, Table 5, Table 6, Table 7).

## Results

### Pyramidal cells in layer 2

#### Subjective observation (Fig. 1A)

The apical dendrites of PCs in layer 2 differed mainly in the bifurcating point along the apical dendrite where the tufts began to form: distal (L2PC_A) or proximal (L2PC_B). Those with a tuft bifurcating proximally also formed a more extensive dendritic tuft than those that bifurcated more distally. In addition, several L2_PCs had no typical apical dendrites, instead, had an inverted big dendrite towards deep layers, which were named layer 2 inverted PC (L2_IPC).

**L2_TPC:A** (layer 2 tufted PC_A): vertically projecting apical dendrites, distal onset of a tuft formation, forms a small tuft, multiple oblique dendrites before tuft formation.

**L2_TPC:B** (layer 2 tufted PC_B): vertically projecting apical dendrite, proximal onset (often within layer 2) of a tuft formation, forms a broader extensive tuft, multiple oblique dendrites before tuft formation.

**L2_IPC**: (layer 2 inverted PC): vertically inverted apical dendrite projecting to deep layers towards white matter, a relatively proximal or distal onset of a tuft formation, forms a relatively extensive tuft, multiple oblique dendrites.

The apical dendrites of both L2_TPC:A and L2_TPC:B types reached the pia of cortex. Very rarely, PCs looking similar to L2_TPC:A were encountered in layer 1 (named **L1_TPC**), which seemed to have “accidently” displayed there. The apical dendrites of these PCs often projected at an angle rather than simply vertically, and a main axon projected towards white matter with a few minor collaterals emerged out, which appeared similar to some of the atypically oriented layer 2 PCs in the juvenile rat neocortex as reported previously (van Brederode et al., 2000).

### Pyramidal cells in layer 3

#### Subjective observation (Fig. 1B)

The apical dendrites of layer 3 PCs commonly formed a tuft distally, which differed mainly in the number of oblique dendrites, either multiple (L3PC_A) or none to a few (L3PC_B) oblique dendrites.

**L3_TPC:A** (layer 3 tufted PC_A): vertically projecting apical dendrites, distal (occasionally proximal) onset of tuft formation, forms a small (occasionally extensive) tuft, multiple oblique dendrites before tuft formation.

**L3_TPC:B** (layer 3 tufted PC_B): vertically projecting apical dendrites, distal onset of tuft formation, forms a small tuft, no or a few oblique dendrites before tuft formation.

The apical dendrites of both L3PC types reached the pia of cortex.

### Neuromorphometric description (Table 2, Table 3)

Quantitative analysis was based on 3D reconstructions of three types of layer 2 PCs (L2_TPC:A, n = 6; L2_TPC:B, n = 33; L2_IPC, n = 4), and two types of layer 3 PCs (L3_TPC:A, n = 35; L3_TPC:B, n = 9).

#### Soma

The soma surface area of L2_TPC:B was significantly larger than L2_TPC:A. There was not significant difference in the perimeter and the surface area between the types of layer 3 PCs.

#### Basal dendrites

The types of L2_PCs and L3_PCs virtually shared similar basal dendritic features respectively. Within each layer, there was no significant difference in the measurements of basal dendrites examined except that L2_TPC:B had significantly higher number of segments than L2_TPC:A. Their basal dendrites consisted of 4–5 dendritic trees with an average branch order of 4 per tree and a max branch order of 5–6. However, compared cross the two layers, the basal dendritic clusters of L3_TPCs were on average bigger than L2_TPCs (P < 0.05) as evidenced by the increased measurements in the max horizontal extends (230 ± 6 μm *vs*. 188 ± 6 μm), the total lengths (2406 ± 119 μm *vs*. 1907 ± 119 μm) and surface areas (5795 ± 434 μ m^2^ *vs*. 4333 ± 366 μ m^2^) and volumes (1415 ± 166 μ m^3^ *vs*. 1014 ± 125 μ m^3^), and segment lengths (54 ± 1 μm *vs*. 46 ± 2 μm) and surface areas (126 ± 7 μ m^2^ *vs*. 100 ± 5 μ m^2^) and volumes (39 ± 3 μ m^3^ *vs*. 22 ± 2 μ m^3^).

#### Apical dendrite

The big broad extensive apical dendrites of the L2_TPC:Bs made several measurements significantly higher than the apical dendrites of L2_TPC:As in (Table 2), including the total apical length, surface area and volume, and segment number. On average, L2_TPC:As, however, had a significantly higher number of oblique dendrites (6.5 ± 1.1) than L2_TPC:Bs (5.0 ± 0.4). Interestingly, L2_IPCs tended to have the highest number of oblique dendrites (8.5 ± 1.9). In contrast, the simple apical dendrites of L3_TPC:Bs made several measurements significantly lower than the apical dendrites of L3_TPC:As (Table 3), including the maximum horizontal extent, total length, surface area and volume, segment number, maximum branch order. On average, L3_TPC:As also had more oblique dendrites (4.9 ± 0.3) in comparison with L3_TPC:Bs (2.3 ± 0.4).

Compared cross layers, although the apical dendrites of L3_PCs were vertically longer (L3_PCs: 346 ± 11 μm *vs*. L2_PCs: 231 ± 8 μm), L2_PCs (L2_TPC:A & L2_TPC:B) had broader apical dendrites (the maximum horizontal extent, L2_PCs: 239 ± 12 μm *vs*. L3_PCs: 177 ± 8 μm), longer total length (2488 ± 146 μm *vs*. 1980 ± 113 μm) and higher number of segments (47 ± 5 *vs*. 35 ± 2). The apical dendritic clusters of L2_PCs were on average ∼1.35 fold wider and ∼1.29 fold longer than those of L3_PCs. Therefore, L2_PCs, particularly the L2_TPC:Bs, have a more complex apical dendritic cluster compared with L3_PCs.

With their inverted apical dendrites L2_IPCs are similar to inverted PCs found in layer 6 (L6_IPC). The apical dendrites of L2_IPCs are about to quantitatively compare with the L6_IPCs in the layer 6 PC section below.

#### Axon

In comparison with L2_TPC:A, the L2_TPC:B showed a significantly larger axonal extent, total length and surface area, number of segments as well as the maximum branch order. This suggested that the L2_TPC:Bs may have denser local axonal clusters near the soma. The axons of L3_PC types were not significantly different. The density of boutons along the axon was similar in L2_PCs and L3_PCs, ranging from 18 to 21 boutons/100 μm on average.

Previous studies have pooled L2 and L3 PCs, yielding two types, which primarily differ in axonal morphology in mouse SSC (Larsen and Callaway, 2006). One type is typical for layer 2/3 PCs, sending axonal minor collaterals into layers 3 and 5 avoiding layer 4 (*i.e*., type I 2/3 PC in that study). The other type as a minor group is usually located at the border of layer 3 and has significantly more axonal minor collaterals distributed in layer 4 (*i.e*., type II 2/3 PC). Some L3_TPC:As in the current study look similar to the type I 2/3 PC and the L3_TPC:B looks similar to the type II 2/3 PC in that previous study. However, local axonal projections may vary depending upon different cortical areas. In the auditory cortex, L2/3 PCs have substantial axonal arbors in layer 4 as well as in layers 3 and 5 (Barbour and Callaway, 2008). Furthermore, excitatory inputs to L2/3 PCs received within a functional column seem all similar in the primary visual and somatosensory and auditory cortices since these PCs receive strong excitation from layers 2 and 4 (Larsen and Callaway, 2006; Barbour and Callaway, 2008).

### Spiny neurons in Layer 4

#### Subjective observation (Fig. 1C)

Spiny neurons in layer 4 of the rat SSC were clearly identified into three types based on the characteristic features of apical dendrites – tufted (L4_TPC), untufted PCs (L4_UPC) (L4_TPC & L4_UPC together named L4_PCs) and stellate cells (L4_SSC).

**L4_TPC** (layer 4 tufted PC): vertically projecting apical dendrite, distal onset of tuft formation, forms a small tuft, multiple oblique dendrites before tuft formation.

**L4_UPC** (layer 4 untufted PC): vertically projecting apical dendrite, no tuft formation, multiple oblique dendrites emerged proximally in most cases.

**L4_SSC** (layer 4 spiny stellate cell): vertically projecting apical-like dendrite more frequently branching but having a radial length not much longer than basal dendrites, no tuft formation, forms multiple oblique dendrites fewer than those of L4_PCs.

The apical dendrites of the three spiny neuron types in layers 4 typically did not reach layer 1, occasionally, reaching the inner half of layer 1.

#### Neuromorphometric description (Table 4)

Quantitative analysis was based on 3D reconstructions of the three types of layer 4 neurons (L4_TPC, n = 44; L4_UPC, n = 33; L4_SSC, n = 12)

### Soma

L4_SSC had smaller somata than L4_TPC and L4_UPC. Sizes of somata of L4_TPC and L4_UPC were similar.

### Basal dendrite

On average, L4_TPCs had 6 basal dendrites while L4_UPC and L4_SSC types had 5 basal dendritic trees. All 3 types of L4 spiny neurons had an average of 3 branch orders per dendritic tree with a maximum branch order of 5. The basal dendrites of L4_TPCs appeared to have the longest total length, which was significantly longer than those of L4_UPCs. Compared with the L4_TPC and L4_UPC, L4_SSC was characterized by curved basal dendritic segments as indicated by a significantly higher tortuosity. Furthermore, the total surface area and volume of the basal dendrites of L4_SSCs appeared the smallest among the three types of L4 spiny neurons, suggesting that the basal dendrites of a L4_SSC may receive less synaptic inputs.

### Apical dendrite

The apical dendrites of all three L4 spiny neuron types were vertically oriented towards the pia. However, the vertical extent of L4_SSCs’ apical dendrites (191 ± 14 μm) was significantly shorter – only 32% and 45% of the extents of L4_TPCs (547 ± 23 μm) and L4_UPCs (451 ± 23 μm), respectively. Further quantification of the maximum horizontal extent, total length, surface area, volume, segment number, and maximal branch order of the apical dendrites demonstrated that the size of an apical dendrite was the biggest in L4_TPCs, intermediate in L4_UPCs and the smallest in L4_SSCs. Similar to the basal dendrites, the apical dendrites of L4_SSCs also had notably curvier segments. L4_SSCs had an average of 4.5 oblique dendrites, significantly less than the oblique dendrite number of two types of L4_PCs (averagely 6.4 oblique dendrites per cell).

### Axon

Despite the fact that axonal minor collaterals of PCs were severed due to the preparation of brain slices, the axons of L4_SSCs appeared significantly different from the two L4_PC types. The total length and surface area and volume as well as the segment number of the L4_SSC axon were significantly greater than those of the two L4_PC types. But the average length of axonal segments of L4_SSCs was significantly shorter. These quantitative results together represented a rich local axonal cluster, corresponding to the L4_SSCs’ locally denser axonal cluster that primarily remains within one column (Feldmeyer et al., 1999; Staiger et al., 2004). In addition, the branch angles were significantly different among the three types of L4 spiny neurons, indicating different axonal branch patterns of individual types.

In a previous study using thicker brain slices (500 μm thick), three anatomical subclasses of layer 4 excitatory neurons, largely corresponding to the three types identified in the current study, have been defined (Staiger et al., 2004). As reported, the spiny stellate cells (L4_SSCs) confine their axonal arbors to the local microcircuit of their origins. Since more axonal minor collaterals are obtained from thicker slices, the difference between the axonal clusters of other two types becomes more evident. The pyramidal neurons, corresponding to the L4_TPCs, have many transcolumnar branches extending into neighboring microcircuits; the star pyramidal cells (L4SPCs, corresponding to the L4_UPCs), have axonal arbors showing both a columnar component and transcolumnar branches containing the highest bouton density. Consistent with this previous study, the bouton density of L4_UPCs (22 ± 1 boutons/100 μm) was significantly higher than those of L4_TPCs and L4_SSCs (19 ± 1 and 18 ± 1 boutons/100 μm; both P = 0.02) in the current study.

### Pyramidal cells in layer 5

#### Subjective observation (Fig. 1D)

L5_PCs were identified into four types based on the characteristic features of apical dendrites. Three of them were tufted types, which were further identified according to the tuft size, and the bifurcating pointsproximally or distally along the apical dendrites.

**L5_TPC:A** (layer 5 thick-tufted PC_A): vertically projecting apical dendrite, distal onset of tuft formation, forms a broad thick tuft, multiple oblique dendrites emerged proximally.

**L5_TPC:B** (layer 5 thick-tufted PC_B): vertically projecting apical dendrite, proximally bifurcating into two and each further distally bifurcate into smaller tufts (conjointly forming a thick tuft), multiple oblique dendrites emerged proximally.

**L5_TPC:C (**layer 5 small tufted PC): vertically projecting apical dendrite, distal onset of tuft formation, forms a small tuft, multiple oblique dendrites emerged proximally.

**L5_UPC (**layer 5 untufted PC): vertically projecting apical dendrite, no tuft formation, forms multiple oblique dendrites emerged proximally in most cases.

The apical dendrites of L5_TPC:As and L5_TPC:Bs reached the pia, whereas those of L5_TPC:C and L5_UPC often reached only layer 4 or up to supragranular layers of cortex.

#### Neuromorphometric description (Table 5)

The quantitative analysis was based on 3D reconstructions of the four types of layer 5 PCs (L5_TPC:A, n = 60; L5_TPC:B, n = 38; L5_TPC:C, n = 33; L5_UPC, n = 30).

### Soma

As evidenced by the bigger perimeter and surface area, L5_TPC:A and L5_TPC:B types had significantly bigger somata than those of L5_TPC:C and the L5_UPC.

### Basal dendrites

The horizontal extent of basal dendritic clusters of layer 5 PC types was approximately equivalent to the width of a local cortical microcircuit (∼300 μm as defined in previous studies (Jones, 1983; Favorov and Diamond, 1990; Land et al., 1995; Lubke et al.,2003)), except L5_UPCs that had narrower basal dendritic cluster. L5_TPC:A and L5_TPC:B were bigger neurons, which had a basal dendritic cluster consisting of 7 basal dendritic trees on average. L5_TPC:C and the L5_UPC were smaller neurons, which had a basal dendritic cluster consisting of 6 dendritic trees. Similarly, L5_TPC:A and L5_TPC:B types had a max branch order of 6 yielding one more compared with two small L5PC types that have a max branch order of 5. All types of layer 5 PCs had an average of 4 branch orders per dendritic tree except of L5_TPC:C yielding 3 branch orders on average.

Compared with the small types (*i.e*. L5_TPC:C and the L5_UPC), the basal dendrites of large L5_PCs (*i.e*., L5_TPC:A and L5_TPC:B) were significantly greater in the total length, surface area and volume and the number of segments, but shorter in segment length. This implies that the basal dendrites of large L5_PCs are constructed with a higher number of shorter and thicker segments while the small L5_TPC:Cs and the L5_UPCs are constructed with a lower number of longer and thinner segments. Large L5PCs have, therefore, a significantly greater basal dendritic surface to receive synaptic input in comparison with the two small types.

### Apical dendrites

The maximum horizontal extent of apical dendrite was wider than the width of a cortical column (∼300 μm) in the two large L5_PC types (L5_TPC:A, 356 ± 10 μm; L5_TPC:B, 350 ± 12 μm), but narrower in the two small types (L5_UPC, 216 ± 10 μm; L5_TPC:C, 252 ± 11 μm). Compared with the small types, the apical dendrites of large L5_PCs were significantly greater in the total length and surface area and volume and the number of segments.

The horizontal extent of L5_UPC apical dendrite was significantly narrower than that of L5_TPC:C, which was the narrowest among all the layer 5 PCs. The total length and surface area of L5_TPC:B apical dendrite was significantly greater than that of L5_TPC:A, and the total length of L5_TPC:C apical dendrite was significantly longer than that of L5_UPC. Interestingly, the L5_TPC:C apical dendrite had the longest average segment length, which was significantly longer than those of L5_TPC:A and L5_TPC:B. The L5_TPC:C apical dendrite also tended to have a lower number of oblique dendrites. A neuron subpopulation similar to L5_TPC:C type has been previously described according to the specific appearance of the apical dendrite as well as the layer-specific axonal arborization and expressing a high level of a transgenic marker protein in mouse cortex (Akemann et al., 2004; Larsen and Callaway, 2006; Larsen et al., 2007).

### Axon

The axons of L5_TPC:A and L5_TPC:B shared similar morphological properties except the tortuosity and branching angles. The tortuosity value of the L5_TPC:B axon was the lowest among all layer 5 PC types, consistent with the basal and apical dendrites of this type. The axon of the L5_TPC:B is, therefore, constructed with relatively straight segments all over different compartments. In addition, L5_TPC:A (15 boutons/100 μm) and L5_TPC:B (16 boutons/100 μm) had bouton densities significantly lower than those of L5_TPC:C and L5_UPC (both: 21 boutons/100 μm). Bouton densities were similar between the two large L5_PC types and between the two small L5_PC types, respectively.

Retrograde labeling of single neurons *in vivo* with recombinant rabies virus has made it possible to reconstruct the complete axonal structure of layer 5 PC types and reveals clear differences in local axonal clusters for different types in the mouse barrel cortex (Larsen et al., 2007). The thick-tufted PCs (corresponding to the L5_TPC:A and L5_TPC:B in the current study) project their local axons within deep cortical layers, while the slender-tufted PCs (corresponding to the L5_TPC:Cs) and the short untufted PCs (corresponding to the L5_UPCs) have extensive projections to superficial layers. The axons of L5_UPCs are relatively columnar, while those of L5_TPC:Cs have extensive laterally spreading with patchy arborization within layer 2/3. A study using retrograde labeling of single neurons in rat vibrissal cortex with *in vivo* patch-clamp recording and full morphological reconstruction reports that axons of L5_UPCs are about 2.7 fold longer than large L5_PCs (Oberlaender et al., 2011). In the current study, PCs were reconstructed from 300 μm thick brain slices, where the laterally spreading axonal processes have been largely severed during the slicing procedure. Compared against *in vivo* labeling, morphological measurements obtained by *in vitro* labeling were obviously underestimated, particularly with respect to the maximum axonal extent, segment number, the total and segment length, surface area and volume.

### Pyramidal cells in layer 6

#### Subjective observation (Fig. 1E)

The L6_PCs had the most diversified morphologies of apical dendrites, which granted a classification of as many as six PC types.

**L6_TPC:A** (layer 6 tufted PC_A): vertically projecting apical dendrite, distal onset of tuft formation, forms a small tuft, multiple oblique dendrites.

**L6_TPC:C** (layer 6 tufted PC_C or ***Narrow PC***): a narrow-looking TPC - vertically projecting apical dendrite, distal onset of tuft formation, forms a small tuft, often more oblique dendrites than other PC types.

**L6_UPC** (layer 6 untufted PC): vertically projecting apical dendrite, no tuft formation, multiple oblique dendrites.

**L6_IPC** (layer 6 inverted PC): vertically inverted apical dendrite projecting towards white matter, distal onset of tuft formation, forms a small tuft, multiple oblique dendrites.

**L6_BPC** (layer 6 bitufted PC): vertically projecting apical dendrite, distal onset of tuft formation, forms a small tuft, multiple oblique dendrites. In addition, a big inverted dendrite often slightly obliquely projecting downwards, distal onset of tuft-like formation, forms a small tuft-like plexus, multiple oblique dendrites.

**L6_HPC** (layer 6 horizontal tufted PC): horizontally projecting apical dendrite, distal onset of tuft-like formation branching into a few tuft branches, forms a few oblique dendrites.

The apical dendrites of layer 6 PCs often reached the layer 4 or supragranular layers, but very rarely reached layer 1. L6_TPC:As and L6_UPCs could be termed *typical PCs* because of the similarity of their apical dendrites with the TPC and UPC types in other layers. The remaining types of PCs were specific for layer 6, and identified by distinct morphologies of their apical dendrites.

The L6_TPC:C type corresponds to the corticothalamic cells that have been extensively characterized among all layer 6 PCs more recently with optogenetic techniques (Olsen et al., 2012; Bortone et al., 2014; Kim et al., 2014; Crandall et al., 2015). At a first glance, L6_TPC:Cs resembled L6_TPC:As, but had notably narrower overall structures (also named *narrow PCs*), which were composed of a small basal dendritic cluster, a narrow apical dendrite and a cluster of predominant vertical axonal minor collaterals directed towards the pia. L6_TPC:Cs typically had a small tuft reaching layer 4 or 5, rarely layer 1. Their axons projected towards white matter with a main axonal collateral while gave out minor collaterals projecting upwards within a cortical columnar range, barely horizontally projecting towards distant cortical regions. In contrast, the horizontally extending minor axonal collaterals were common for all other types of PCs in layer 6.

L6_IPCs had no typical upward apical dendrite, instead, a big dendrite inverted towards the white matter and branching more frequently than a typical basal dendrite. They also had a particular axonal initiation, either at the side of the soma facing the pia, subsequently looping and extending downwards, or at an inverted primary dendrite with certain distance away from the soma. These morphological features were consistent with previous reports (Mendizabal-Zubiaga et al., 2007).

L6_BPCs had a typical apical dendrite oriented towards the pia, with or without a small tuft and a big inverted dendrite oriented vertically or obliquely towards the white matter that branched more often than a typical basal dendrite, resulting in a ‘bipolar’ somatodendritic appearance. L6_BPCs have been reported in a few previous studies (Katz, 1987; Zhang and Deschenes, 1997).

The apical-like dendrites of L6_HPCs were not typically oriented upwards, but extended horizontally with a couple of more branches than other basal dendrites.

Interestingly, different types of PCs were found almost all oriented obliquely or even horizontally in the bottom part of the layer 6 (corresponding to layer 6b) (data is not included due to small samples).

#### Neuromorphometric description (Table 6)

The quantitative analysis was based on 3D reconstructions of the six types of layer 6 PCs (L6_TPC:A, n = 26; L6_TPC:C, n = 18; L6_UPC, n = 23; L6_IPC, n = 27; L6_BPC, n = 32; L6_HPC, n = 7).

### Soma

The somata of L6_HPCs appeared to be the biggest and were significantly different from other PC types in layer 6. The somata of other types were not significantly different from each other in both perimeter and surface area.

### Basal dendrites

The basal dendrites of L6_TPC:Cs were unique among the layer 6 PCs in that they comprised the lowest maximum horizontal and vertical extensions and segment lengths, but contained the highest number of dendrites that were very tortuous. The maximum horizontal extent was about as wide as only half of a cortical column width. Correspondingly, the total and segmental length, surface area and volume of L6_TPC:C basal dendrites were the smallest among all layer 6 PCs. Therefore, the basal dendritic cluster of L6_TPC:C consists of a higher number of small narrow trees with short, tortuous segments.

In high contrast, L6_HPCs appeared to be another unique type, having the biggest basal dendritic cluster among all PCs in layer 6. The dendritic extents of L6_HPCs were 1.6–2.5 fold horizontally, and 1.7–2.9 fold vertically larger than other types. The maximum horizontal extent of the L6_HPCs was wider than the width of a typical cortical column (*i.e*., 300 μm). In addition, the basal dendrites of L6_HPCs were characterized by the lowest dendritic tree number and tortuosity, and smaller branch angles. Consequently, the basal dendritic cluster of a L6_HPC consisted of a few large trees with long, straight segments.

With the exception of L6_TPC:Cs and L6_HPCs, other layer 6 PC types had on average 5–6 basal dendrites per neuron, although the L6_BPC had 4 dendrites on average plus a big inverted one counted as an inverted apical-like dendrite.

The basal dendrites were almost the same between L6_TPC:As and L6_UPCs, with a significantly smaller local spline angle in the latter. Taken together, the total dendritic length of L6_TPC:As and L6_UPCs were greater than all other layer 6 PCs, except HPCs.

### Apical dendrites

Consistent with the basal dendrites, L6_TPC:Cs also had a unique apical dendrites, which was the narrowest among all layer 6 PCs, with the highest maximum branch order, tortuosity as well as the highest number of oblique dendrites. Together, these features represented a narrow apical dendrite of L6_TPC:Cs with many curvy oblique and tuft branches.

The apical arbors of L6_HPCs were largely consistent with the features of their basal counterparts, having the widest maximum horizontal extent, longest segment length and the lowest number of segments with the lowest maximum branch order.

Despite the notable difference in the tuft, quantitative measurements of apical dendrites were similar in L6_TPC:As and L6_UPCs, except the maximum horizontal extent and the number of oblique dendrites. L6_TPCs had a wider maximum horizontal extent with a higher number of oblique dendrites and a wider maximum horizontal extent that appeared due to the tuft structure.

The apical dendrites of all layer 6 PCs in the SSC mostly reached layers 4 and 5, occasionally reaching layers 2 and 3, and almost never reaching layer 1.

Compared with the L2_IPCs (Table 7), the inverted dendrites of L6_IPCs were bigger as evidenced by significantly greater horizontal and vertical extends and longer segment length. But the basal dendrites of the two types of inverted PCs were very similar in almost all measured parameters except that the basal dendrites of L2_IPCs had more branches.

### Axon

In contrast with highly diversified dendritic morphologies, quantitative analysis of the axons of all layer 6 PCs in brain slices demonstrated that they appeared largely similar, with the exception of L6_TPC:Cs. Consistent with the basal and apical dendrites, L6_TPC:C also had the narrowest axonal cluster as evidenced by the smallest maximum horizontal extent approximately equaling to the width of a cortical column. Correspondingly, the maximal and planar angles of L6_TPC:C axons were significantly bigger than those of other layer 6 PCs. In addition, the density of boutons along the axon appeared to be the lowest in L6_TPC:Cs (17 boutons/100 μm) and the highest in L6_HPCs (22 boutons/100 μm), significantly different between these two. Otherwise, the bouton density was similar among the other types of layer 6 PCs, ranging from 19 to 20 boutons/100 μm on average.

## Discussion

Different morphological classes of cortical PCs have characteristic properties in intrinsic electrophysiology and synaptic innervations in both local and distal neuronal networks (Thomson, 2010). The PCs in infragranular layers have been studied most intensively.

Layer 5 PCs distinguished by the morphology of their apical dendrites have distinctive projection targets as reported previously (Schofield et al., 1987; Hallman et al.,1988; Hubener and Bolz, 1988). Layer 5 PCs that contain thick apical dendrites with prominent terminal arbors in layer 1 (corresponding to the large L5_PCs or L5_TPC:A and L5_TPC:B in the current study) project to subcortical targets including the superior colliculus *via* the cerebral peduncle, the pontine nuclei, the pretectal area, the thalamic matrix, and to the striatum. Neuron types with shorter and untufted apical dendrites (corresponding to the L5_UPCs) project to the contralateral cortex. In an *in vivo* study on intracortical pathways in vibrissal cortex for whisker motion and touch, the functional differences of large and small types of L5_PCs have been examined in behaving rats (Oberlaender et al., 2011). Large types of L5_PCs reliably increase spiking preferably after passive touch while small types of L5_PCs carry motion and phase information during active whisking, but remain inactive after passive whisker touch. Although the large types of L5_PCs appear to share the same long-range projections to subcortical targets (Larsen et al., 2007), the L5_TPC:A and L5_TPC:B types have clearly been distinguishable not only in their morphology but also in their electrophysiological properties and synaptic physiology in rat medial prefrontal (Wang et al., 2006) and sensorimotor cortices (Franceschetti et al., 1998). In the ferret prefrontal cortex, L5_TPC:A neurons are characterized by a single thick-tufted apical dendrite, exhibit accommodating firing of action potentials (AP), and are interconnected with depressing synapses. Whereas, L5_TPC:B neurons are distinguished by dual apical dendrites, display non-accommodating AP discharge patterns, and are hyper-reciprocally connected with facilitating synapses displaying pronounced synaptic augmentation and post-tetanic potentiation. It appears that L5_TPC:A and L5_TPC:B neurons form distinct synaptic sub-networks respectively within the local prefrontal neocortex (Wang et al., 2006). Sub-networks composed of homogenous PCs have also been reported in the layer 5 of rodent neocortex (Le Be et al., 2007; Brown and Hestrin, 2009). Comparatively less intensively studied, the projections of layer 5 PCs with a small tuft (corresponding to the L5_TPC:Cs) have been reported only in a few studies (Akemann et al., 2004; Larsen and Callaway, 2006). Using a retrograde tracer with recombinant rabies virus to fill full-structures of layer 5 PCs, it was found that L5_TPC:C like cells project to contralateral targets (Larsen et al., 2007). In another study, two groups of L5_TPC:C-like neurons projecting to the striatum and corpus callosum, respectively (Hattox and Nelson, 2007). Callosal L5_TPC:C-like cells have significantly shorter apical dendrites and are usually found in the upper part of layer 5 (corresponding to layer 5a).

Layer 6 PCs project strongly to the thalamus, the claustrum, other ipsilateral cortical areas and the contralateral hemisphere (Briggs, 2010; Thomson, 2010). An *in vivo* tracing study reported that the somatic, dendritic and axonal morphology reliably predict the main projection targets of the axon, enabling a classification of layer 6 PCs according to their long-range projections (Zhang and Deschenes, 1997). Cortico-cortical cells (CCs), that have ipsilateral long-range axonal minor collaterals and project callosally to the other cortical hemisphere, have a big inverted primary dendrite (corresponding to L6_IPCs) or an apical dendrite (corresponding to typical PCs including L6_TPC:As and L6_UPCs). Cortico-thalamic cells (CTs), projecting to the specific and/or unspecific thalamic nucleus, also have an apical dendrite, but the axonal arborisation within the cortex is spatially confined, not much wider than the extent of its apical dendrite (corresponding to narrow PCs, *i.e*., L6_TPC:Cs). Similar cell types of CT and CC neurons have also been reported in another study, in which the CT cells correspond to narrow PCs (L6_TPC:Cs) and the CC1 and CC2 cells correspond to L6_TPC:As and L6_UPCs respectively (Kumar and Ohana, 2008). Most claustral (CL) projecting neurons have two major dendrites, an apical and a big basal dendrite (corresponding to L6_BPCs). In recent years, CTs have been extensively studied using transgenic labeling techniques combined with optogenetics (Olsen et al., 2012; Bortone et al.,2014; Kim et al., 2014). The narrower appearance of CTs in both dendritic and axonal clusters has been reported as the most striking feature different from all other types of excitatory neurons in layer 6 (Olsen et al., 2012). Injections of fluorescent retrograde tracer *in vivo* into multiple subcortical and cortical axon-target regions revealed that L6_TPC:Cs were specifically thalamus projection neurons while other layer 6 PCs have multiple distant projecting targets (unpublished data).

Interestingly, different types of layer 6 PCs are also distinguishable in their intrinsic and synaptic dynamic properties (Thomson, 2010). Electrophysiologically, both CCs and CLs display powerful spike frequency adaptation while CTs display a weakly adapting firing in a near tonic firing pattern; In terms of the synaptic dynamics in the local neuronal circuits, CCs innervate other pyramids much more frequently and stronger than CTs do; CCs and CLs frequently innervate other PCs, but very rarely innervate interneurones, contrasting the case that CTs rarely innervate other PCs, but frequently innervate interneurones in layer 6. A combination of transgenic and optogenetic approaches has demonstrated that layer 6 plays an important role in gain control of synaptic transmission across cortical layers (Olsen et al., 2012; Bortone et al., 2014), and also differentially modulates neuronal activity in different cortical layers (Kim et al., 2014). The function of this gain control is based on the synaptic innervation from CTs (corresponding to narrow PCs or L6_TPC:Cs in the current study) to fast spiking inhibitory neurons (*i.e*., basket cell family, BCs) in layer 6 as well as in other layers (Olsen et al., 2012; Bortone et al., 2014). CTs differentially modulate synaptic activity in different layers (Kim et al., 2014), forming facilitating synapses on PCs and BCs and Martinotti cells in layer 5, and PCs in layer 6, but depressing synapses on BCs in layer 4 (unpublished data, also see (Beierlein and Connors, 2002; West et al., 2006; Crandall et al., 2015). The anatomical features of narrow somatic, dendritic and axonal morphologies could endow CTs with the specificity for the signal processing within a primary cortical column, which can be evidenced by the exquisite tuning of the activity of CTs to orientation and direction information (Velez-Fort et al., 2014; Grieve and Sillito, 1995). By virtue of being the largest neuronal population consisting of about 65% the total excitatory cells in layer 6 (Olsen et al., 2012) and having the ability to fire APs at high frequencies (Mercer et al., 2005), CTs could be actively involved in cortical processing by converging facilitating and depressing synaptic inputs onto postsynaptic cells (Beierlein and Connors, 2002; Crandall et al., 2015).

In the study on the granular layer of the somatosensory cortex, L4_SSCs have been distinguished from L4_PCs distributed together within barrel columns, whereas only L4_PCs are distributed in the septa regions between barrel columns (Brecht and Sakmann, 2002). L4_SSCs and L4_PCs show different synaptic properties even within the same barrel column. L4_SSCs show strong responses with almost constant amplitudes *in vivo* to stimulation of principal whiskers, whereas L4_PCs depress subsequently although showing an initial amplitude similar to L4_SSCs and the postsynaptic responses of septum-PCs are initially much weaker and depress subsequently (Brecht and Sakmann, 2002). Consistent with these results, as revealed by patch clamp recording of monosynaptic connections from brain slices, L4_SSCs form strong synaptic connections almost exclusively with neurons located within the same barrel (Markram et al., 1997; Feldmeyer et al., 1999; Petersen and Sakmann, 2000; Brecht and Sakmann, 2002; Schubert et al., 2003). These results indicate that L4_SSCs function predominantly as local signal processors within single barrels, which is basically determined by their dendritic and axonal structures restricted within a barrel column. By contrast, L4_PCs (including L4_TPCs and L4_UPCs) connect with neurons not only within the same barrel column but also from neighboring barrels (Schubert et al., 2003).

In terms of the afferent thalamocortical innervation, L4_SSCs receive input signals from the VPM nucleus (Diamond et al., 1992), and are more strongly influenced by thalamocortical synaptic input than other PC types in layer 4 (Benshalom and White, 1986; Brecht and Sakmann, 2002; Staiger et al., 2004), while L4_PCs in septa receive afferent input from axons originating in the PoM nucleus (Koralek et al., 1988; Chmielowska et al.,1989). Having greater dendritic surface area and denser local axonal clusters around somata, L4_SSCs as a majority population in layer 4 could form an efficient local synaptic network, which is fundamental to amplify weak thalamic inputs and relay thalamocortical signals for information processing across different layers within the same barrel column. On the other hand, L4_PCs could form weaker but broader synaptic networks to input from sources within and outside the same barrel column to synchronize network activity across barrel columns. This capability of L4_PCs depends upon their dendritic and axonal structures that often extend into multiple barrel columns and septa. According to previous studies, L4_PCs predominantly give out commissural and associative axonal collateral projections (Wise and Jones, 1976; Code and Winer, 1986; Lewis and Olavarria, 1995), suggesting the involvement of these L4_PC types in the network activity at a whole brain level.

The correlation between specific cell types of layer 4 and long-distance projections has been reported in a study on primary visual cortex (V1) of macaque monkey with injections of a fluorescent protein expressing rabies virus into the middle temporal (MT) or the secondary visual cortex (V2) (Nassi and Callaway, 2007). It was found that L4_SSCs of V1 are the majority of neurons projecting to MT, and L4_PCs are the majority of those projecting to V2.

Compared with PCs in infragranular and granular layers, the correlations between specific populations of cell types and local and long-distance afferent inputs or efferent projections have been less extensively studied in the supragranular layers of neocortex. According to previous studies, L2_PCs are involved in cross-columnar integration ensembles, whereas L3_PCs participate in intracolumnar circuits in sensory cortices (Schubert et al., 2007; Staiger et al., 2015). In terms of the afferent innervation, L2_PCs and L3_PCs preferentially receive different thalamocortical input from POm and VPM respectively (Meyer et al., 2010). L2_PCs are likely to receive POm input on their apical tufts and probably lack VPM input, whereas L3_PCs receive strong input from the VPM on their basal and apical oblique dendrites, and apical tufts. It can be expected that a wider apical architecture is crucial for L2_TPC:B to not only contribute to cross-columnar information processing but also to provide a broad apical domain to receive input from POm.

In accordance with the fact that the long-range axonal projection of PCs is an important feature useful for the classification of cortical and subcortical principle neurons (Larsen and Callaway, 2006; Larsen et al., 2007; Boudewijns et al., 2011; Aransay et al., 2015), in recent years, new approaches combining different optical imaging techniques and long-range axon labeling with transgenic techniques and virus injections have been gradually developed, which make it possible to reconstruct single neurons with long-range axonal projections at whole brain level (Yuan et al., 2015; Economo et al., 2016; Gong et al., 2016). Although it is not yet sufficient to make a systematic study of PC classification in any specific cortical region so far, these approaches are useful for more accurate identification and differentiation of single or multiple long-range axonal projections and for a quantitative mapping of distal projecting targets of those PC types that can be sparsely labeled. It is expectable that a brain atlas of rodent animals will be eventually built at a single neuron resolution in future, which would be fundamental for biologically detailed simulations of neuronal microcircuitry of brain regions and ultimately of the whole brain (Markram, 2006).

As the last to be addressed, the composition of PC populations as well as the somatodendritic morphological features of individual PC types in each layer may change at varying degrees depending upon the developmental stage, the functional cortical region and the species of animals (Jacobs and Scheibel, 2002; Elston, 2007; Spruston, 2008a,b; Elston and Fujita, 2014; Elston and Manger, 2014; Luebke, 2017). In terms of development stages, pyramidal cells in the cortex have developed with relatively complex, highly branched basal and apical dendritic structures after a fast overall growth before the age P14 (Wise et al., 1979, Romand et al., 2011). At the subsequent stages to adults, it is featured with the slow localized growth by thickening mainly on intermediates or lengthening mainly on terminals accompanied by the retraction on different segments (Romand et al., 2011). While the proposed approach in the current study largely holds true for basic PC classes across primary sensory cortices, more complicated diversity of somatodendritic morphologies has indeed been revealed in different primary cortical regions. For instance, L3_PCs having an early bifurcating apical dendrites without tuft formation are found in primary visual cortex of monkey (Rockland, 1992); The apical dendrites of a layer 6 PC type frequently reach layer 1 in the visual cortex (Olsen et al., 2012). Furthermore, the somatodendritic structures, especially the apical dendrites, of PCs in higher-order association regions become more complex, resulting in the composition of PC populations significantly different from primary cortical regions (Wang, Markram et al., 2006; van Aerde and Feldmeyer, 2015; Kawaguchi, 2017). In the prefrontal cortex where no layer 4 exists, all PCs in layers 2, 3 and 5 have a tufted apical dendrite. Especially, those having a broad tufted are found at a rate of 100% in layer 2, 55% in layer 3 and 27% in layer 5 respectively. Even in layer 6, 33% of PCs form a simple tuft in layer 1 (van Aerde and Feldmeyer, 2015). On the other hand, afferent and efferent diversification can result in multiple sub-divisions of the same morphological type of PCs within the same layer. For instance, morphologically indistinguishable PCs in the same cortical layers have been found to receive different inputs and send different outputs (Akemann et al., 2004; Larsen and Callaway, 2006; Feldmeyer, 2012). This kind of complicated neuronal diversity would be better explored with molecular techniques such as single cell transcriptomics (Poulin et al., 2016; Tasic et al., 2016), which is out of the discussing category of the current study.

## Conflict of interest

The Authors declare no conflict of interest.
